# High levels of indoor fine particulate matter during the cold season in Almaty prompt urgent public health action

**DOI:** 10.1371/journal.pone.0285477

**Published:** 2023-05-04

**Authors:** Denis Vinnikov, Venerando Rapisarda, Sergey Babanov, Ermanno Vitale, Leonid Strizhakov, Zhanna Romanova, Irina Mukatova

**Affiliations:** 1 Occupational Health Risks Laboratory, RUDN University, Moscow, Russian Federation; 2 Environmental Health Laboratory, al-Farabi Kazakh National University, Almaty, Kazakhstan; 3 Occupational Medicine, Department of Clinical and Experimental Medicine, University of Catania, Catania, Italy; 4 Department of Clinical Pharmacology and Occupational Disease, Samara State Medical University, Samara, Russian Federation; 5 Department of Internal, Occupational Diseases and Rheumatology, Sechenov First Moscow State Medical University, Moscow, Russian Federation; 6 Laboratory of Workers’ Reproductive Health Disorders Prevention, Izmerov Research Institute of Occupational Health, Moscow, Russian Federation; 7 Department of Internal Diseases, Lomonosov Moscow State University, Moscow, Russian Federation; 8 Department of Epidemiology, Biostatistics and Evidence-Based Medicine, al-Farabi Kazakh National University, Almaty, Kazakhstan; 9 Department of Internal Diseases with Courses of Nephrology, Hematology, Allergology, and Immunology, Astana Medical University, Astana, Kazakhstan; Satyawati College, University of Delhi, INDIA

## Abstract

**Introduction:**

Almaty is the largest city of Kazakhstan with extreme air pollution, mostly in the cold season, but little is known whether staying indoors could lessen the exposure. The aim was to quantitatively characterize indoor fine PM levels and to verify the contribution of ambient pollution to it in a polluted city like Almaty.

**Methods:**

We collected forty-six 24-hour 15-min average samples of the ambient air and a similar number of paired indoor samples (total 92 samples). Predictors of both ambient and indoor PM_2.5_ mass concentrations in mg/m^3^, including ambient concentration, precipitation, minimal daily temperature and humidity, along with the indoor/outdoor (I/O) ratio were tested in the adjusted regression models at eight 15-min lags.

**Results:**

Ambient air PM_2.5_ 15-min average mass concentrations were highly variable and ranged from 0.001 to 0.694 mg/m^3^ (geometric mean (GM) 0.090, geometric standard deviation (GSD) 2.285). Snowing was the strongest predictor of lower ambient PM_2.5_ 24-hour mass concentrations (median 0.053 vs 0.135 mg/m^3^ (p<0.001)). Indoor mean 15-min PM_2.5_ concentrations ranged from 0.002 to 0.228 mg/m^3^ (GM 0.034, GSD 2.254). In adjusted models, outdoor PM_2.5_ concentration explained 0.58 of all variability of the indoor concentration with a 75-min delay (R^2^ 0.67 at lag8 on snowing days). Median I/O ranged from 0.386 (IQR 0.264 to 0.532) at lag0 to 0.442 (IQR 0.339 to 0.584) at lag8.

**Conclusion:**

During the cold season when fossil fuel is burnt for heating, the population in Almaty is exposed to very high fine PM levels even indoors. Urgent public health action is needed.

## Introduction

Almaty, the largest city of Kazakhstan with a population of 2 million inhabitants, has been reported a place of extreme air pollution, mostly in the cold season [1]. Almost 100% of average daily samples in the cold season exceed the current daily exposure limit of fine particulate matter (PM), PM_2.5_ of 0.035 mg/m^3^, reflecting the use of colossal amount of fossil fuel for heating both at the centration power plants and by the private households, whereas exposure levels remain within the limit most of the time in summer. As recent study shows, population is exposed to detrimental levels of fine PM in the cold season, including those staying outdoors most of the time [2]. That personal exposure analysis not only demonstrated very high PM_10_ mass concentrations (PM_10_ median 0.352 mg/m^3^) in the air, but a significant variation in exposure, making it difficult to comprehensively assess the associated health effects. In response to devastating air pollution levels in the cold season, a number of monitoring and notification resources is available to the public; however, the reported fine PM concentrations do not tend to improve.

Despite the concern over public health consequences of dramatic air pollution in Almaty, yet little is known about the contribution of selected sources to that pollution, and the views of the government, research community and environmental activists differ. Valid scientific methodology to verify the contribution of fossil fuel combustion for heating in the households, coal combustion for heating and power generation at the centralized generating facilities and car exhausts has to be elucidated. Given that air quality dramatically improves in summer, burning coal for heating both by the power stations and private sector is likely the leading source of pollution in the city [1]. In addition, the population stays indoors most of the time during the cold season, if not employed for the outdoor workplaces, and their exposure also remains unknown. Infiltration quantification could shed some light to indoor exposure levels, but such studies have never been published from Kazakhstan.

Moreover, the infiltration coefficient is largely affected by a wide range of variables, including, but not limited to, the year of construction, ventilation, outdoor temperature, window opening, indoor cooking, number of inhabitants, etc. [3–5]. A recent study from China demonstrated that PM_2.5_ infiltration factor was mostly affected by the season, air conditioner use and meteorological factors, but the variation remained wide [6]. The study conditions were very heterogenous, when the time of window opening differed, as did the air conditioner use and time of cooking. Compared to Almaty, reported air pollution levels in such studies were much lower (the median of 0.077 mg/m^3^ in the study [6]) and the outdoor temperatures were significantly higher. Indoor air pollution levels in Almaty with its extreme outdoor fine PM levels during the heating season with windows persistently closed has never been analyzed. We, therefore, aimed to quantitatively characterize indoor fine PM levels in a highly polluted city in the cold season and to verify the contribution of ambient pollution to it.

## Materials and methods

### Venue

Almaty is the largest city of Kazakhstan with a population of 2 million people and situated in the Southeast of the country. Historically, this has been an industrially developed region within the economic system of the Soviet Union, and a fairly wide range of industries employed most population. Plants and factories together with private and public automobiles contributed most to air pollution, in contrast with formerly developed network of zero emission buses (trolleybuses) and trams. However, after the demolition of the Soviet Union, when all heavy industry was terminated, air pollution was still gradually worsening, and recently, PM pollution was reported very high, almost extreme, in the city during the cold season, November through March [2]. This is likely due to low rate of natural gas use in the suburbs, when a large fraction of population in the suburbs may use coal, wood and even tires, plastic and dung for heating in the cold season. Pollution with PM in Almaty is summer, including months from May through September, remains within the exposure limits set by the government and is of no public health concern, but increases 10-20-fold in winter [1], which includes December, January and February, coinciding with the heating season. Therefore, air pollution is a pronounced public health issue in Almaty during the cold season only.

Most of the population within the city traditionally resides in apartment blocks, all heated centrally with hot water supplied by three large power plants, which operate on coal, and smaller plants spread all over the city using natural gas. In addition, apartment blocks of nine floors and less use natural gas for cooking, whereas taller buildings are equipped with electric ovens. Therefore, there is no coal combustion in apartment blocks of any year of construction. In contrast, private residential houses in the city and in the suburbs may have gas supply, but may combine its use with coal or decline gas use completely, depending on the economic level of the household. The fraction of exhausts from three power plants, private households and automobiles continue to be a matter of debate [1].

### Ambient and indoor PM_2.5_ measurements

**Fig 1:**
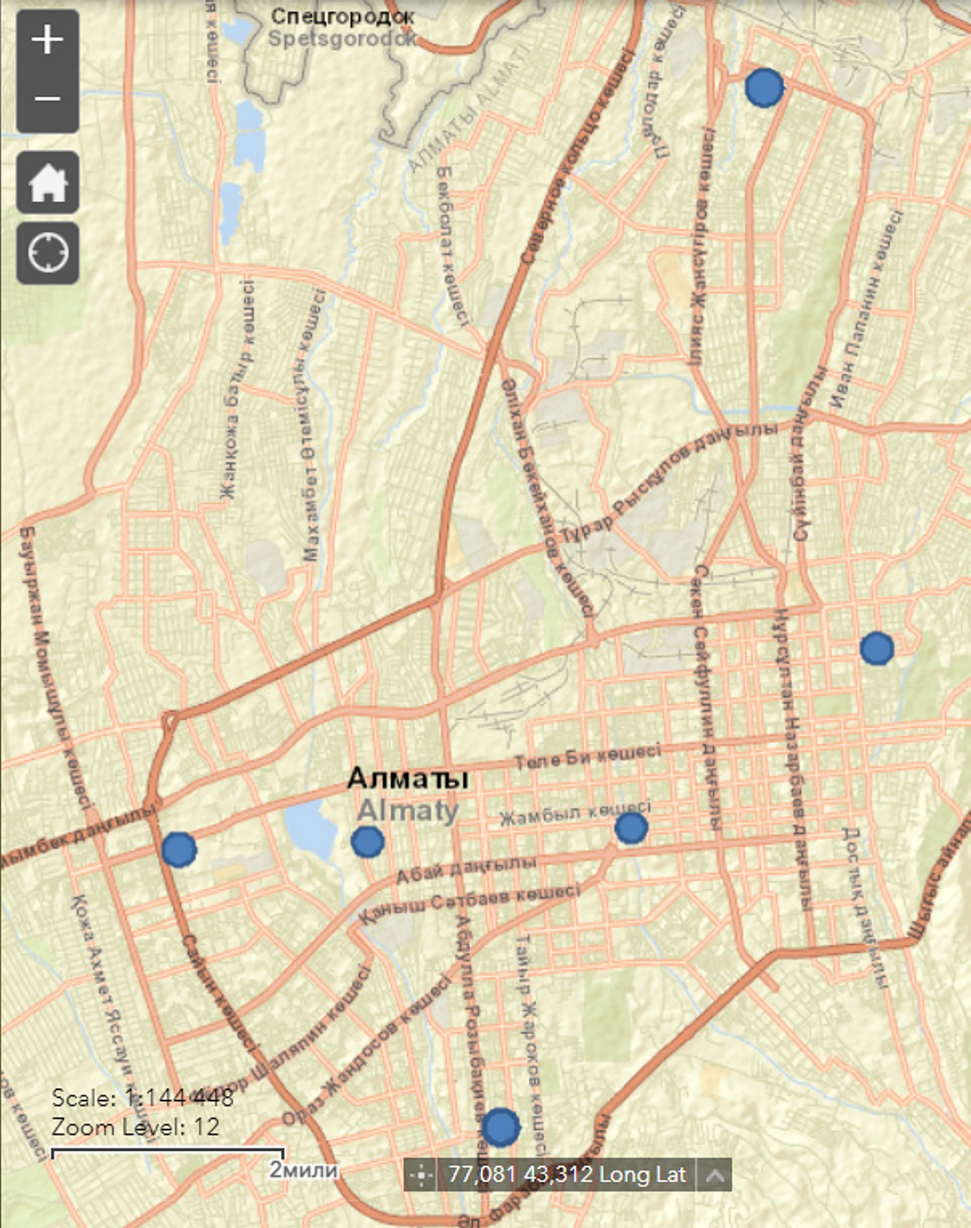
Locations of apartments included in the study. Map from http://viewer.nationalmap.gov/viewer/ and is for illustrative purposes only.

We randomly chose six typical apartments, two of which were situated in 9-storey buildings; two more in 5-storey buildings and the remaining two were in 2-storey houses made of reinforced concrete in Almaty. All six houses were located all over the city, embracing the southern, northern, western and eastern parts and the city center (Fig 1). Apartment blocks were built from 1974 to 2009. Permission from all house owners to collect samples was obtained. Exposure variability of the indoor sources, such as window opening and cooking is well-documented [5–7], whereas the contribution of the ambient pollution in its high concentrations to the indoor levels remains poorly described; therefore, we minimized the indoor emissions by no cooking in the apartment and keeping windows closed. Air conditioners were not used. There was only one person living in all six apartments throughout the observation period.

We used two TSI SidePak AM520 direct-reading instruments in this study. Both devices were calibrated timely by the producer and zeroed daily prior to measurements. The device uses light-scattering technology to measure and report mass concentration in mg/m^3^ of selected PM separated with an impactor. Internal pump is calibrated to provide constant air flow with 1.7 l/min. Device for ambient measurements was placed outdoors 50 cm away from the building, whereas the indoor measurements device was placed in one of the living rooms at an elevation of 1 m above the floor. Both devices were turned on and off simultaneously, providing paired PM_2.5_ measurements for 24 hours every day through the study period. We set logging intervals to 15 minutes, thus yielding 15-min average points of 1-sec measurements. These 15-min mass PM_2.5_ concentrations were arithmetic means of 900 1-sec data.

In addition, we extracted daily minimal and maximal ambient air temperatures, mean daily relative humidity, mean daily wind speed and precipitation from www.gismeteo.ru for precise location of the apartments.

### Statistical analysis

We did not aim to characterize infiltration factor, as this would had required to measure outdoor and indoor sulfur concentrations assuming that such PM would likely have outdoor origin. However, because windows opening, cooking indoors and the use of air conditioning were not allowed, we implied that most indoor PM had the outdoor origin. In addition, indoor pollution would likely be explained by the infiltration of the outdoor PM inside the building because ambient PM concentrations in Almaty in the cold (heating) season were very high, as demonstrated before [1, 2]. We also recently demonstrated that indoor respirable PM concentrations even in the beauty salons were mostly determined by high outdoor concentrations [8]. We, therefore, set the indoor to outdoor (I/O) ratio as the primary endpoint in our study. That was calculated as the ratio of indoor PM_2.5_ mass concentration at a given timepoint to outdoor concentration at the same timepoint (lag0), to outdoor concentration 15 minutes ago (lag0), to outdoor concentration 30 min ago (lag1) and all the way to lag8, which corresponded to 8*15 minutes = 2 hours or 120 minutes. Secondary endpoints were PM_2.5_ ambient and indoor mass concentrations.

All concentrations were recorded as 15-min average, grouped to daily averages and analyzed in descriptive procedures in terms of distribution normality. Because all data, including indoor and outdoor concentrations, were left-skewed, we used medians with the corresponding interquartile ranges (25^th^ to 75^th^ percentiles) (IQR) to describe data. Similarly, we used non-parametric tests to verify differences in the univariate two-group comparisons, such as snowing with non-snowing days (Mann-Whitney U-test) and whether between-group variance exceeded the one between groups when comparing several groups (Kruskall-Wallis test), such as days of observation. We tested whether ambient concentrations were associated with precipitation, min and max daily air temperatures, relative humidity and wind speed using simple linear regression models. We found that precipitation was a strong predictor of exposure; therefore, we also compared all concentrations on snowing with those on non-snowing days in two-group comparisons. In addition, daily means were also compared against 24-hour exposure limit for ambient air set in the Republic of Kazakhstan (0.035 mg/m^3^), and the number of days when the concentration stayed within the limit was recorded.

Given that the association of measured indoor PM_2.5_ mass concentrations with the outdoor levels followed linear pattern, we applied linear regression models to test this association. In the univariate linear regressions, precipitation, minimal daily temperature, and humidity, but not maximal temperature or wind speed were found associated with the indoor exposure levels. Therefore, adjusted models included outdoor concentrations, minimal daily temperature, precipitation and humidity as predictors of the current indoor PM_2.5_ concentration (lag0), with 15-min delay (lag1) and so on to 2-hours delay (lag8). We report beta coefficients of the outdoor levels with the corresponding 95% confidence intervals (CI) and the overall R^2^ for the model. Furthermore, we stratified such models into those including only snowing days and, separately, non-snowing days. P-values were reported for group comparisons. All tests were completed in NCSS 2021 (Utah, USA) (https://www.ncss.com/software/ncss/procedures/)).

## Results

During the cold season of December 2021 and January 2022, we collected forty-six 24-hour 15-min average samples of the ambient air and additionally a similar number of paired indoor samples (total 92 samples) with the overall sampling time 1104 hours or 4416 paired 15-min data points. Ambient air minimal day temperatures ranged from -9 to +7 degrees C (median -2; IQR -4;0), apparently lower than the air maximal day temperatures, which ranged from -3 to +11 degrees C (median +4; IQR +1;+6). Average daily relative humidity during the study period ranged from 36 to 91% (median 61%; IQR 51;76%), significantly greater on days with precipitation, median 80% vs. 57%. Out of the total of 46 days of observation, it snowed on nine days (20% of days). The average daily wind speed ranged from 1 to 4 (median 2; IQR 2;3) m/s (Table 1).

**Table 1:** Temperatures, relative humidity and wind speed on the days of observation.

	Min daily temperature, C	Max daily temperature, C	Relative humidity, %	Wind speed, m/s
Min	-9	-3	36	1
Max	+7	+11	91	4
GM	1.676	4.406	60.764	2.172
25^th^percentile	-4	1	51	2
50^th^ percentile (median)	-2	4	61	2
75^th^ percentile	0	6	76	3

Note: GM–geometric mean

**Fig 2:**
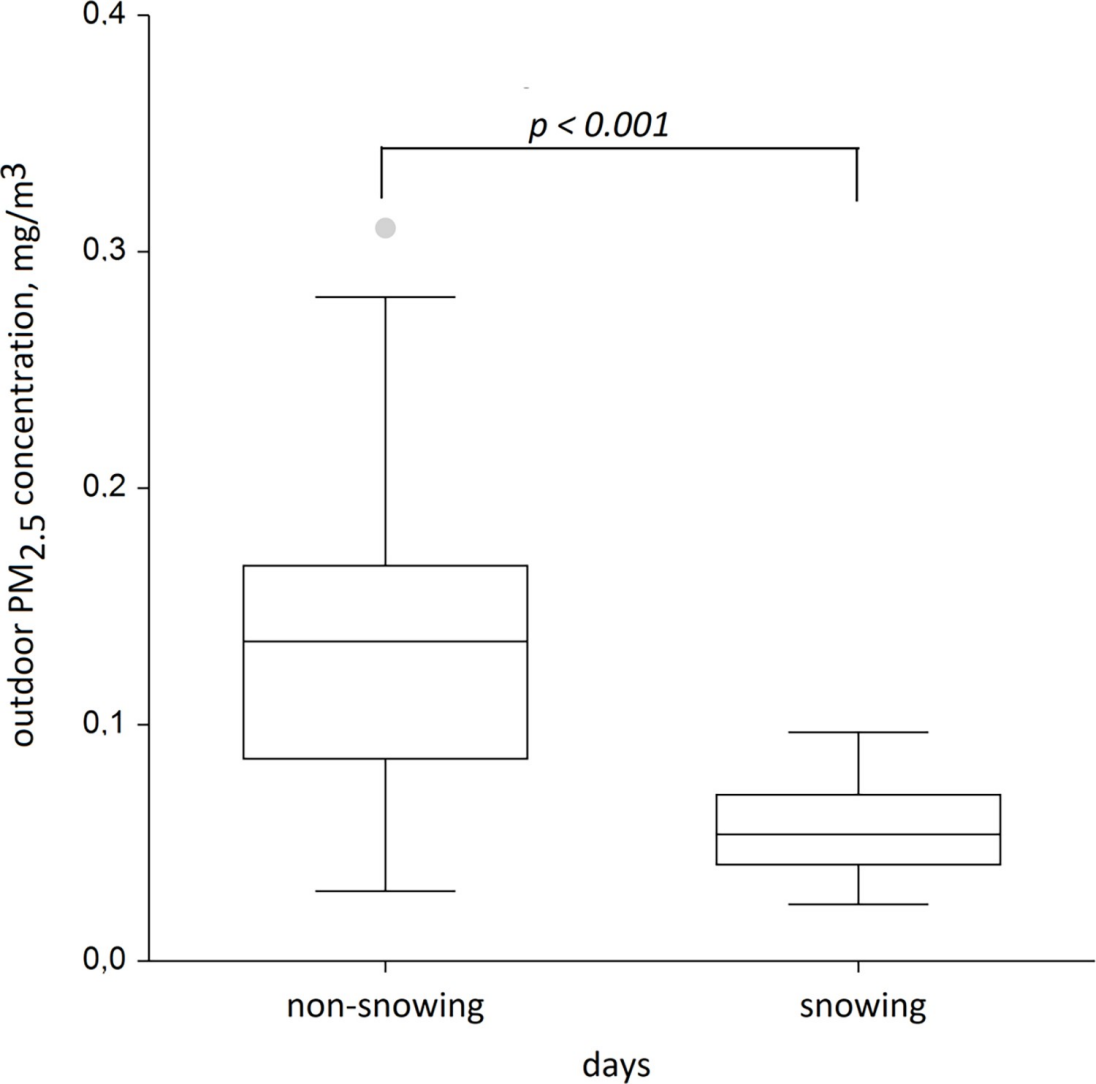
Median and interquartile range of outdoor PM2.5 mass concentrations on snowing and non-snowing days.

Ambient air PM_2.5_ 15-min average mass concentration demonstrated very high variability and ranged from 0.001 to 0.694 mg/m^3^ with a median of 0.092 (IQR 0.055; 0.166) mg/m^3^. Geometric mean was 0.090 mg /m^3^. Daily 24-hour means ranged from 0.024 to 0.286 mg/m^3^ with a median of 0.104 (IQR 0.066; 0.146) mg/m^3^. Given that the current exposure limit for the 24-hour mean ambient concentration in Kazakhstan was 0.035 mg/m^3^, we observed only two days out of forty-six when 24-hour mean PM_2.5_ outdoor air concentrations were below exposure limit (4% days). In a Kruskall-Wallis test, between-day variance was significantly greater compared to within-day variances (p<0.001), indicative of significant differences in PM_2.5_ mass concentrations between the days. Precipitation (snowing) was the strongest predictor of lower ambient PM_2.5_ 24-hour mean mass concentrations, when the corresponding median concentrations on the snowing and non-snowing days were 0.053 and 0.135 mg/m^3^ (p<0.001) (Fig 2).

**Fig 3:**
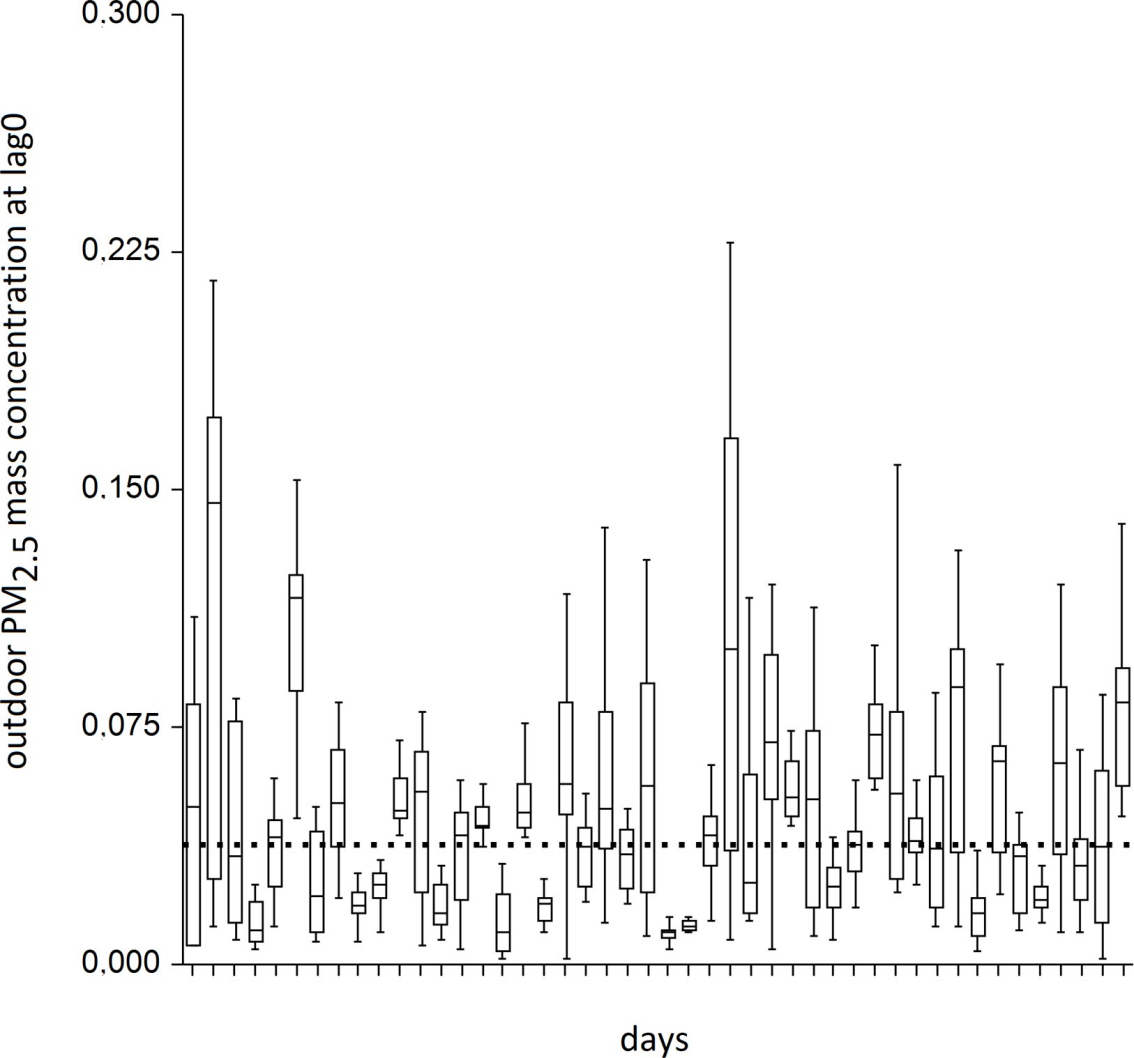
Median, interquartile (IQR) and IQR*1.5 (whiskers) indoor PM_2.5_ concentrations on all studied days. Dotted line is the median concentration of all 15-min measurements.

Indoor mean 15-min PM_2.5_ concentrations ranged from 0.002 to 0.228 mg/m^3^ with the median 0.038 (IQR 0.020;0.060) mg/m^3^. As with the outdoor concentrations, we found highly significant differences in between-days comparison using Kruskall-Wallis test (p<0.001) (Fig 3). Out of 46 days studied, we found daily median concentrations below the median of all measurements (0.038 mg/m^3^) in 21 days (43% days). Because there was no indoor exposure limit in Kazakhstan, we compared daily mean levels with the World Health Organization (WHO) air quality guideline levels, which equaled 0.015 mg/m^3^ for PM_2.5_ during 24 hours. Thus, on only 4 of 46 days studied (9% days), the mean daily concentration was 0.015 mg/m^3^ or less. Snowing also affected indoor concentrations, decreasing the mean 15-min concentrations two-fold (median 0.021 vs. 0.045 mg/m^3^, p<0.001).

**Fig 4:**
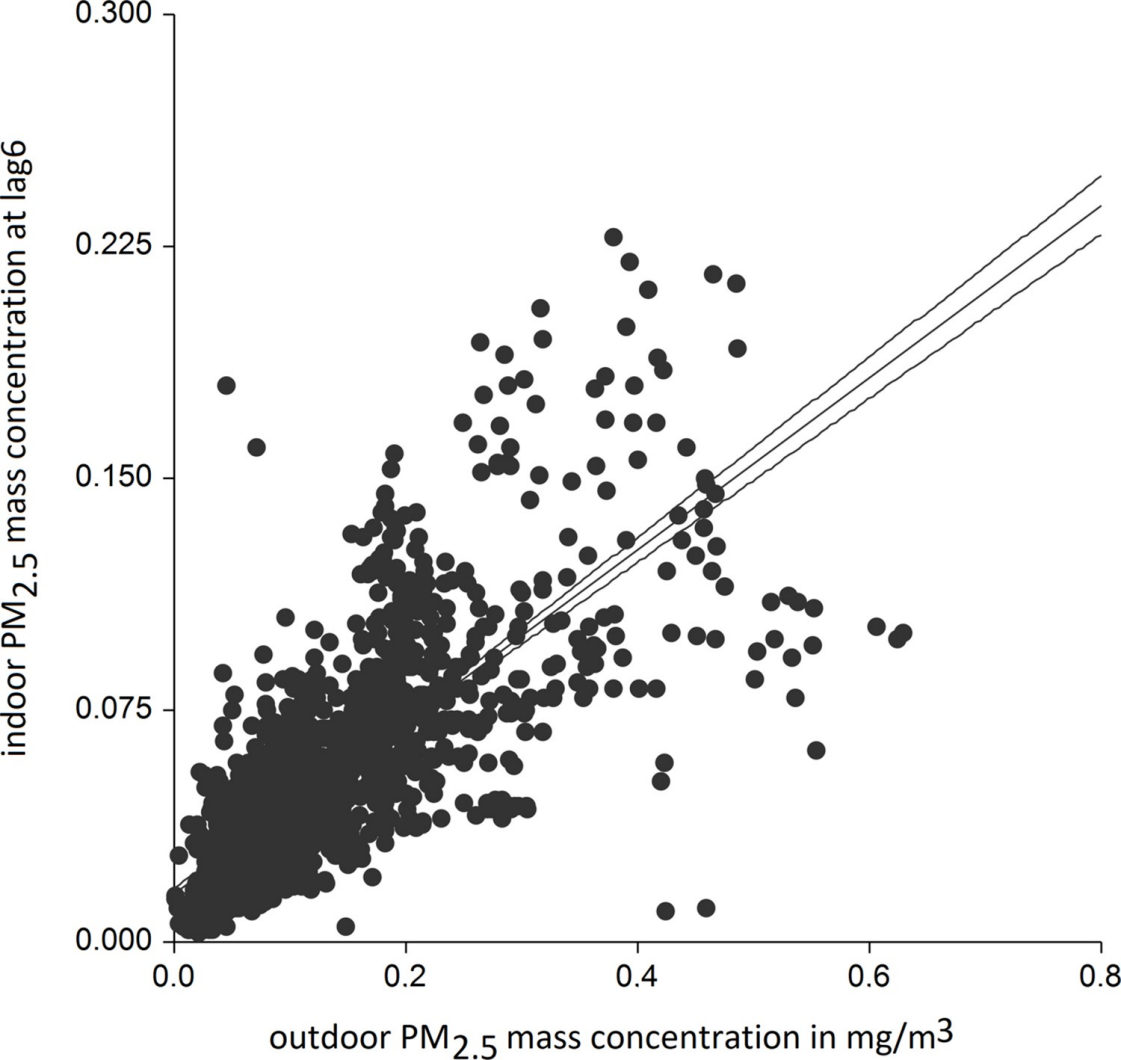
Scatter plot of indoor PM_2.5_ mass concentrations vs. outdoor concentrations at lag6 on all days (R^2^ of adjusted model 0.58). Prediction line is with 95% confidence intervals.

**Fig 5:**
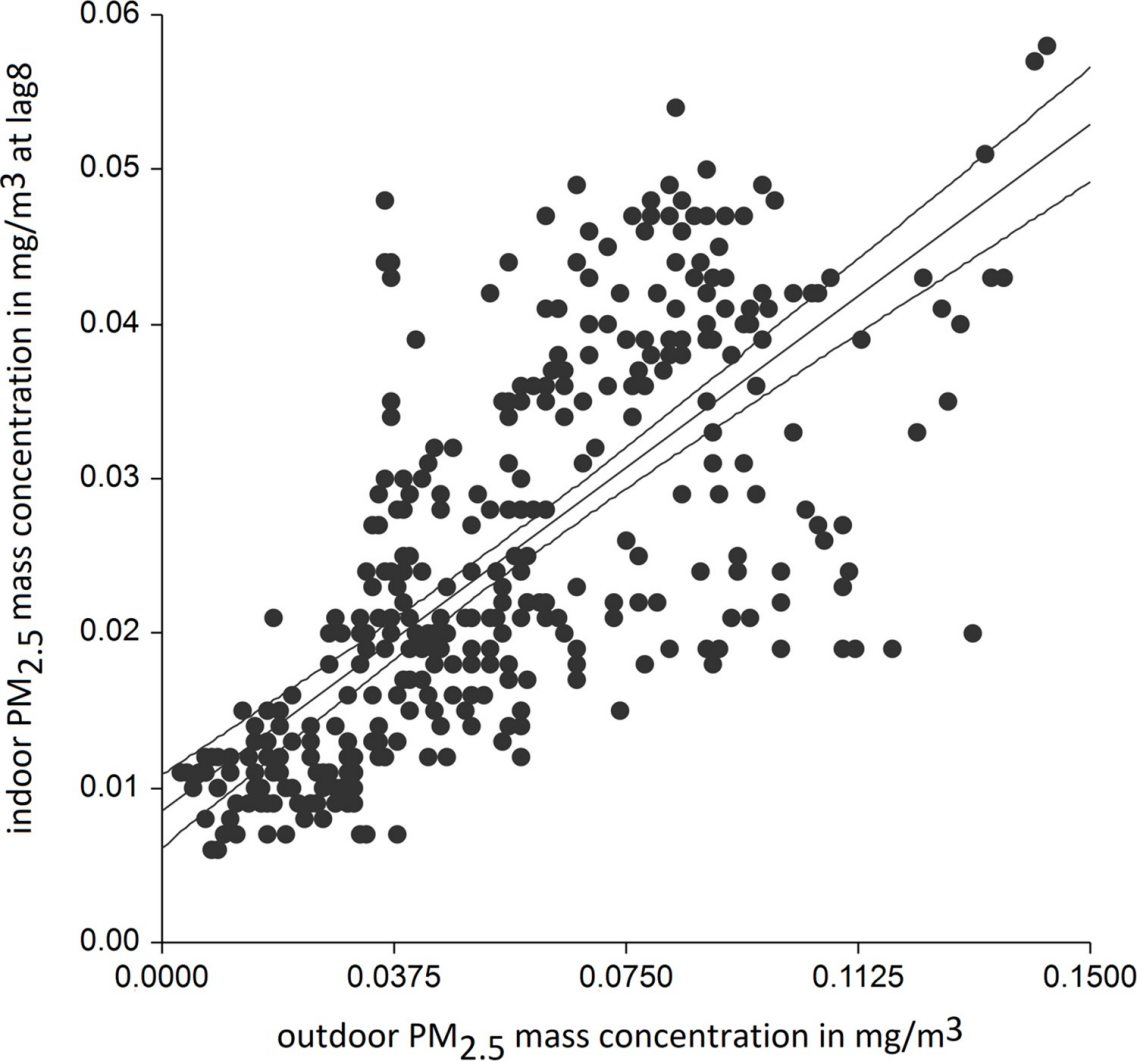
Scatter plot of indoor PM2.5 mass concentrations vs. outdoor concentrations at lag8 on snowing days (R^2^ of adjusted model 0.67). Prediction line is with 95% confidence intervals.

The outdoor PM_2.5_ level (Table 2) was the strongest predictor of indoor PM_2.5_ mass concentrations with the highest R^2^ and power of all tested variables. We found time-dependent association between these two variables, when the highest R^2^ was reached after 1 hour and 15 minutes (lag5). In adjusted for precipitation, humidity and minimal daily ambient temperature models, outdoor PM_2.5_ concentration explained 0.58 of all variability in the indoor concentration with 1 hour and 15 min delay (Fig 4). Moreover, when we stratified the models into snowing and non-snowing days, we found that the outdoor concentration was even a stronger determinant of the indoor level on snowing days, when the overall concentrations were significantly lower both outdoors and indoors (R^2^ = 0.67 at lag8) (Fig 5) (Table 2). Of note, in all adjusted models including stratified into snowing and non-snowing days, the outdoor least temperature was also a significant predictor of indoor PM_2.5_ concentrations.

**Table 2:** Regression beta coefficients of the indoor PM_2.5_ concentrations for outdoor PM_2.5_ mass concentrations and the corresponding R^2^ for models in adjusted models.

	Lag0	Lag1	Lag2	Lag3	Lag4	Lag5	Lag6	Lag7	Lag8
All days									
R^2^	0.48	0.52	0.54	0.56	0.57	0.58	0.58	0.58	0.58
β	0.23	0.24	0.25	0.25	0.26	0.26	0.26	0.27	0.27
β LL	0.22	0.23	0.24	0.24	0.24	0.25	0.25	0.25	0.25
β UL	0.24	0.25	0.26	0.26	0.27	0.27	0.28	0.28	0.28
Snowing days									
R^2^	0.50	0.54	0.57	0.60	0.62	0.63	0.65	0.66	0.67
β	0.22	0.23	0.24	0.25	0.25	0.26	0.26	0.26	0.26
β LL	0.19	0.21	0.22	0.22	0.23	0.23	0.23	0.24	0.24
β UL	0.25	0.26	0.27	0.28	0.28	0.29	0.29	0.29	0.29
Non-snowing days									
R^2^	0.40	0.44	0.44	0.49	0.50	0.50	0.51	0.50	0.50
β	0.22	0.24	0.24	0.25	0.25	0.26	0.26	0.26	0.26
β LL	0.21	0.22	0.23	0.24	0.24	0.24	0.25	0.25	0.25
β UL	0.24	0.25	0.26	0.26	0.27	0.27	0.28	0.28	0.28

Note: LL–lower 95% confidence interval limit; UL–upper 95% confidence interval limit. Beta coefficients are adjusted for T_min_, humidity and precipitation (yes/no)

I/O ratio at lag0 ranged from 0.026 to 6 with severe left-skewness (skewness 5.2), and ratio above 1 was identified on days with very low outdoor exposure only, such as on snowing days. Median I/O ratio equaled 0.386 with IQR from 0.264 to 0.532 at lag0, whereas the geometric mean was 0.376 (Table 2), assuming that at lag0 approximately one-third of the outdoor concentration was registered indoors. A clear trend of increasing I/O with simultaneous increase in data left-skewness was identified, and the largest I/O ratio was registered at lag8, when indoor PM2.5 mass concentration was almost half of what was outdoors two hours before (Table 3).

**Table 3:** I/O ratio at different time points of observation.

Time point	Median	IQR	Skewness
Lag0	0.386	0.264 to 0.532	5.2
Lag1	0.396	0.273 to 0.532	16.8
Lag2	0.400	0.286 to 0.531	16.9
Lag3	0.407	0.294 to 0.537	17.0
Lag4	0.413	0.305 to 0.543	18.1
Lag5	0.421	0.313 to 0.551	18.9
Lag6	0.429	0.323 to 0.560	18.8
Lag7	0.433	0.333 to 0.574	25.8
Lag8	0.442	0.339 to 0.584	26.1

Note: IQR–interquartile range

## Discussion

This is the first report in the available scientific literature on the exposure to PM_2.5_ concentrations in the residential space indoors in the largest and most polluted city of Kazakhstan, Almaty, during the heating season. We now demonstrated that with very high outdoor fine PM concentrations, when PM_2.5_ 24-hour mean may reach 0.286 mg/m^3^, almost as high as in selected workplaces including welding [9] and shish kebab cooking [10], Almaty residents are also exposed to unhealthy levels of fine PM pollution even indoors with windows constantly closed and no cooking allowed. Furthermore, the pattern of changing indoor fine PM levels followed the ambient concentrations with a 1-2-hour delay, whereas the indoor concentrations would reach 38% to 44% of that outdoors. Of note, on snowing days, the population in Almaty can be exposed to significantly lower PM_2.5_ concentrations both outdoors and indoors.

Preceding studies have characterized infiltration from the outdoor pollution along with its predictors elsewhere. In a Chinese study, where all included predictors could explain 60–68% variability and in which they tested season, air conditioner use, windows opening and other minor determinants, the infiltration factor was as high as 0.83, higher and with greater variance during the transition period [6]. In a larger earlier study [11], infiltration factor averaged 0.62, greater during the warm season. As in the former study, air conditioner use and windows opening were the strongest studied predictors of it, and the models predicted only 60% of the variance in 2-week F_inf_. Air conditioning was also widely discussed in other studies [7, 12]. Of note, one of these studies reported no difference in the infiltration factor (0.52 on average) between warm and cold seasons, and the tested models yielded even lower R^2^ (38%); whereas the other confirmed the contribution of air exchange rate [7]. The latter is believed to be associated with window opening. In addition. one more study found that the most important predictor during the cold-season was outdoor temperature [13].

It is believed that the ambient temperature affects infiltration because it influences residents’ behavior, and this mostly relates to windows opening. Because windows opening impacted infiltration so dramatically and because the overall R^2^ in regression models was only around 60%, we designed our study specifically to preclude the influence of window opening and kept them closed for the duration of the study, also given that windows in winter are kept closed in Almaty anyway. Night temperatures in winter in Almaty and perceived and visible pollution levels in Almaty supported such approach, and the population generally avoided keeping windows open, even for a short time. The highest R^2^ reached in our models was 0.67 on snowing days, when the overall concentrations were low. This finding has distinct implications for policy. Thus, in extremely polluted cities like Almaty, population should be made aware that days with precipitation offer an opportunity for more time to spend outdoors with cleaner air, but with all precautions associated with the risk of hypothermia and slipping.

Most studies which offered measured and calculated I/O ratios and infiltration factors, also during the heating season before were conducted in the cities and regions with significantly lower ambient fine PM concentrations compared to Almaty, and thus those F_inf_ could not be extrapolated to the cities in Kazakhstan during the heating season. Such high exposure to gaseous and particulate air pollution in Almaty has devastating effect on population health, including respiratory and cardiovascular effects. We earlier reported that physical component of health-related quality of life in Almaty was low [14], and its association with very poor air quality would likely be confirmed in future studies, yet unpublished. Respiratory and cardiovascular burden of extreme air pollution in Almaty has never been comprehensively assessed, but can allegedly be high, given that 15% of general population complains of clinically relevant respiratory symptoms and only 24% of subjects with verified chronic obstructive pulmonary disease (COPD) in the study were ever told they had COPD [15]. With the I/O ratio which we demonstrated in our study, we conclude that even staying indoors will not warrant healthy levels of fine PM for the population in Almaty, and a significant fraction of associated health effects can be explained even by indoor air pollution during the cold season.

Our findings will guide clear public health policy components for Almaty during the cold season. First and foremost, burning coal and wood for heating and cooking in the suburbs of Almaty poses colossal burden on healthcare. Other studies in large populations have clearly demonstrated that pollution with fine PM is associated with a very wide range of health effects, including emergency room visits and hospitalizations for cerebrovascular disease, even more in vulnerable groups, such as the elderly [16–18], premature mortality due to cardiovascular disease and ischemic heart disease [19–21], COPD incidence and hospitalization with it [22, 23] and even depression [24] and suicides [25]. Given that low- and middle-income countries like Kazakhstan endure the greatest burden of PM_2.5_-related diseases, such as COPD [26], deposition of PM_2.5_ is very high [27] and a very large fraction of ambient fine PM pollution during the cold season is found indoors, we call for urgent mitigation and control action. Our data now demonstrate that there is no place to escape in Almaty in the cold season from extreme air pollution, and a state-scale and governmental efforts must be undertaken to reduce emission from burning coal and wood in order to improve population health.

The strength of this report is a long observation period, when we managed to measure exposure levels in two coldest months. Furthermore, we used similar paired direct-reading calibrated instruments to capture simultaneous outdoor and indoor concentrations. Finally, we reduced indoor concentrations variability by keeping all windows constantly closed and no cooking in order to reduce confounding and better determine the contribution of outdoor pollution and ratio change over time. The limitations of this analysis include measurements in only one, albeit the largest and most polluted city in Kazakhstan; and no sulfur analysis with subsequent infiltration factor calculation. We measured only one pollutant in our study and did not include gaseous and secondary pollutants, which we consider another limitation of our presentation. Nevertheless, our decision to monitor PM_2.5_ only was stipulated by previously published reports that combustion and the resulting very high PM concentrations are the major concern with regard to air quality in Almaty, when gaseous pollutants concentrations very often stay within the normal limits. In addition, we planned to demonstrate the need for public health action, and fine PM is the pollutant most lined to populational health effects.

## Conclusions

In conclusion, this is the first study from the city of Central Asia with extreme air pollution in winter demonstrating that the population is exposed to very high and unhealthy fine PM levels even when staying indoors with all windows closed. Precipitation in winter, including snowing, is associated with much lower exposure to fine PM in Almaty both outdoors and indoors. On average, during the cold season in Almaty with all windows closed, indoor fine PM concentrations will be one-third of what is found outdoors and may increase to almost half within two hours. Keeping the windows closed is an important, but not comprehensive way to mitigate adverse effects of air pollution with fine PM resulting from burning fuel in Almaty in winter.
